# Healthcare costs for young people transitioning the boundary between child/adolescent and adult mental health services in seven European countries: results from the MILESTONE study

**DOI:** 10.1192/bjo.2023.559

**Published:** 2023-09-26

**Authors:** Alastair Canaway, Rebecca Appleton, Larissa van Bodegom, Gwen Dieleman, Tomislav Franić, Suzanne Gerritsen, Giovanni de Girolamo, Athanasios Maras, Fiona McNicholas, Mathilde Overbeek, Moli Paul, Diane Purper-Ouakil, Paramala Santosh, Ulrike Schulze, Swaran P. Singh, Cathy Street, Priya Tah, Bie Tremmery, Helena Tuomainen, Frank C. Verhulst, Dieter Wolke, Jason Madan, for the MILESTONE Consortium

**Affiliations:** Centre for Health Economics at Warwick, Warwick Medical School, University of Warwick, UK; NIHR Mental Health Policy Research Unit, Division of Psychiatry, University College London, UK; Yulius Academy, Yulius Mental Health Organization, The Netherlands; and Department of Child and Adolescent Psychiatry and Psychology, Erasmus Medical Center, The Netherlands; Department of Child and Adolescent Psychiatry and Psychology, Erasmus Medical Center, The Netherlands; Department of Psychiatry, University Hospital Split, Croatia; and School of Medicine, University of Split, Croatia; Unit of Epidemiological Psychiatry and Evaluation, IRCCS Istituto Centro San Giovanni di Dio Fatebenefratelli, Italy; School of Medicine and Medical Science, University College Dublin, Republic of Ireland; and Lucena Child and Adolescent Mental Health Services, St. John of God Community Services, Republic of Ireland; Yulius Academy, Yulius Mental Health Organization, The Netherlands and Clinical Child and Family Studies, Faculty of Behavioural and Movement Sciences, Vrije Universiteit Amsterdam, The Netherlands; Warwick Medical School, University of Warwick, UK and Children and Young People’s Mental Health Service, Coventry and Warwickshire Partnership NHS Trust, Coventry, UK; Child and Adolescent Psychiatry Unit, Centre Hospitalier Universitaire de Montpellier, Saint Eloi Hospital, France; and Team PsyDev, CESP U1018, INSERM, Université de Versailles Saint-Quentin-en-Yvelines, University Paris Saclay, France; Department of Child and Adolescent Psychiatry, Institute of Psychiatry, Psychology and Neuroscience, King's College London, UK; Centre for Interventional Paediatric Psychopharmacology and Rare Diseases, South London and Maudsley NHS Foundation Trust, UK; and HealthTracker Ltd, UK; Department of Child and Adolescent Psychiatry/Psychotherapy, University of Ulm, Germany; Warwick Medical School, University of Warwick, UK; Department of Neurosciences, KU Leuven, Belgium; Department of Child and Adolescent Psychiatry and Psychology, Erasmus Medical Center, The Netherlands; and Department of Clinical Medicine, University of Copenhagen, Denmark; Department of Psychology, University of Warwick, UK

**Keywords:** Mental health services, cost of illness, transition, randomised controlled trial, Europe

## Abstract

**Background:**

The boundary between services for children and adolescents and adults has been identified as problematic for young people with mental health problems.

**Aims:**

To examine the use and cost of healthcare for young people engaged in mental healthcare before and after the child/adolescent and adult service boundary.

**Method:**

Data from 772 young people in seven European countries participating in the MILESTONE trial were analysed. We analysed and costed healthcare resources used in the 6-month period before and after the service boundary.

**Results:**

The proportion of young people engaging with healthcare services fell substantially after crossing the service boundary (associated costs €7761 pre-boundary *v.* €3376 post-boundary). Pre-boundary, the main cost driver was in-patient care (approximately 50%), whereas post-boundary costs were more evenly spread between services; cost reductions were correlated with pre-boundary in-patient care. Severity was associated with substantially higher costs pre- and post-boundary, and those who were engaged specifically with mental health services after the service boundary accrued the greatest healthcare costs post-service boundary.

**Conclusions:**

Costs of healthcare are large in this population, but fall considerably after transition, particularly for those who were most severely ill. In part, this is likely to reflect improvement in the mental health of young people. However, qualitative evidence from the MILESTONE study suggests that lack of capacity in adult services and young people's disengagement with formal mental health services post-transition are contributing factors. Long-term data are needed to assess the adverse long-term effects on costs and health of this unmet need and disengagement.

Child and adolescent mental health services (CAMHS) typically have a ‘service boundary’, where young people transition to adult mental health services (AMHS) or are discharged elsewhere.^1^ This creates challenges, with AMHS typically having higher severity thresholds for care and stricter criteria.^1,2^ A large proportion of young people have poor transition experiences, failing to get the care they need or even disengaging from care completely,^2–6^ when at a critical juncture developing toward independent adulthood.^1,3,4,7^ Therefore, this service boundary has long been identified as a problematic time point in the development of young people with mental health problems.^8^

Recently, transitional mental healthcare has become an area of growing interest, with interventions and measures being developed to support transition.^1,9,10^ Literature exists on the costs of mental health in certain specific contexts. Waldman et al examined the costs of children with psychiatric diagnoses in a sample with a mean age of 12 years.^11^ Others have focused on specific contexts, such as in-patient services,^12^ interventions,^13,14^ costs of hospital admission/stay,^15^ costs of diagnoses^14–16^ or costs associated with specific subgroups of society (e.g. offenders with personality disorders).^17,18^ However little research has focused on costs of mental healthcare before and after transition. Consequently, little is known about healthcare resource use and costs associated with mental healthcare pre- and post-service boundary. Given the different service cultures and approaches to mental healthcare in CAMHS and AMHS,^1^ there are likely significant differences in the type and quantity of healthcare resource being used before and after the boundary.

## Study aims

The primary aim of this paper is therefore to investigate how patterns of healthcare use change across the transition boundary. We seek to answer the following questions:

- What are the costs of healthcare pre- and post-service boundary in young people engaged with mental healthcare?
- How does healthcare use change over this transition?
- What components of healthcare contribute most to costs pre- and post-service boundary?
- Do healthcare costs differ by severity of mental health problems pre- and post-service boundary?
- Are these patterns of healthcare usage consistent across countries?

We examine the resources used either side of the service boundary in terms of in-patient, out-patient and community care (see Supplementary Material available at https://doi.org/10.1192/bjo.2023.559 for contents of each category). We also stratify by severity, as measured by the Health of the Nation Outcome Scales for Children and Adolescents (HoNOSCA), to investigate the relationship between resource use and severity of illness.

## Method

### Data sources

Data was sourced from the MILESTONE study, which included a longitudinal cohort study and a trial of a transition readiness and appropriateness measure across eight European countries (see Singh et al^19^ for further details on recruitment and study design). Participants were recruited from CAMHS sites delivering medical and psychosocial interventions for young people with mental health and/or neuropsychiatric/developmental disorders. To be included, potential participants were young people with a mental disorder defined by the DSM-IV-TR, DSM-5, ICD-10 or ICD-11, receiving CAMHS care, with an IQ ≥ 70 and within 1 year of reaching the CAMHS service boundary. Additionally, because transition decisions were sometimes made after the young people reached the service boundary, we included young people who were up to 3 months older than the service boundary yet met the other criteria.

This study reports data collected during the MILESTONE trial (registration number NCT03013595). The authors assert that all procedures contributing to this work comply with the ethical standards of the standards of the relevant national and institutional committees on human experimentation and with the Helsinki Declaration of 1975, as revised in 2008. All procedures involving human patients were approved by the UK NHS Health Research Authority National Research Ethics Service (reference 15/WM/0052). Written informed consent was obtained from all patients.

The MILESTONE study evaluated a multi-component intervention centred around the transition readiness and appropriateness measure, which aimed to support and guide CAMHS and AMHS clinicians caring for participants approaching the service boundary.^19^ Data were available at baseline (typically in the 6 months before the service boundary) and 15 months later (post-boundary). The primary measure of mental health status was the HoNOSCA, a 13-item measure that includes questions on behaviour, impairments, symptoms and social functioning. It has a maximum score of 52 and a minimum score of 0, with a higher score indicating worse mental health. The HoNOSCA measure was completed by trained researchers following semi-structured interviews with participants. To capture resource use, a Client Service Receipt Inventory (CSRI) was adapted from previous examples in the mental health literature.^20,21^ Participants provided self-reported information via the CSRI on in-patient, out-patient and community care use over the previous 6-month period. Given the multinational nature of the trial, it was necessary to translate the CSRI from English into the appropriate languages and map service configurations within the individual countries to ensure that resource use was captured consistently across countries within the study. This was completed with the aid of the study team across countries. Data were collected via a web-based, secure data-capture system (HealthTracker™, available commercially from HealthTracker UK at https://www.healthtracker.co.uk/).

### Cost assessment

To calculate costs of healthcare usage, we combined information on resource use with unit costs. Resource use on the types and quantities of healthcare services consumed over the preceding 6 months was collected with the CSRI. Costs for healthcare components were estimated by multiplying the resource used with a unit cost for each individual activity. There is little consensus regarding sourcing of unit costs within multi-country studies.^22^ Given the lack of availability of high-quality unit cost sources in all participating countries, we used a one-country pooled costing approach for this analysis. This entailed the identification of unit costs from the UK where high-quality costing resources exist (e.g. National Health Service reference costs), and conversion to country-specific unit costs by using purchasing power parities (adjusted to prices for Belgium in the year 2015, the year the study commenced).

We present resource use descriptively, examining the proportion of young people in contact with healthcare services before and after the service boundary. Pre-boundary HoNOSCA scores are used as a proxy for mental health severity to provide context when interpreting results. For further disaggregation, we split healthcare into three broad elements: in-patient, out-patient and community care (see Supplementary Material for a list of services included in each category). This allowed us to explore how the composition of care changes across the service boundary. We then repeated these comparisons, using monetary costs to examine how these vary within and between countries. These descriptive results are presented for each country, in addition to a pooled total.

To better understand how severity affects cost, we separated each country's young people into low-, medium- and high-severity tertiles based upon their baseline HoNOSCA scores. This allowed the examination of resource use and cost by mental health severity. Additionally, we created a binary variable based on membership of the high-severity tertile. This was used in conjunction with ‘service destination’ data to compare whether there were differences in cost between those who were and those who were not engaged in mental health services, stratified by severity.

### Statistical methodology

We used multiple imputation within the analysis of cost data to account for missing data.^23^ Multiple imputation was conducted at the cost component level to maximise the use of available data, using chained equations and predictive mean matching (k-nearest neighbours = 5) to avoid the risk of implausible values. Country and health utility (derived from the EQ-5D-5L, using the UK value set) were included as covariates within the imputation equation. We conducted 30 imputations of the data to create 30 data-sets, which were then analysed with multiple imputation procedures within Stata version 16 for Windows.^24^ Additionally, we calculated costs based on completed cases only, and also estimated imputed costs separately for control and intervention arms of the trial.

## Results

### Sample information and clinical outcomes

The sample comprised 772 young people from both arms of the trial: 97 from Belgium, 52 from Croatia, 79 from France, 109 from Germany, 190 from Italy, 118 from The Netherlands and 127 from the UK (we exclude Ireland from our analysis as only 21 young people were recruited there). Missingness was prevalent (see Supplementary Material), particularly in the post-boundary variables. Pre-boundary, missing data ranged from 3% (primary diagnosis) to 36% (transition service destination). For cost variables, this ranged from 11% (in-patient costs) to 17% (out-patient costs). Post-boundary, the proportion missing rose considerably, with approximately a third of cost data being missing post-boundary. Exploratory analysis of missingness found that missing cost data post-boundary was not strongly associated with disease severity, with negligible correlation (0.1) between HoNOSCA score pre-boundary and any missing cost data post-boundary. Across all countries, most participants were White (93%). Ethnicity data was missing for 11% of the young people, particularly in France, where there are legal issues with collecting data pertaining to ethnicity/race. The most common primary diagnoses were anxiety and/or depressive disorders (37%), behavioural disorders (35%), psychotic disorders (9%), feeding/eating disorders (7%), and trauma and stressor-related disorders (7%). Heterogeneity of case mix existed across countries. The UK was dominated by anxiety and depressive disorders (54%), with only 18% having behavioural disorders. Germany was similar, with 44% reporting anxiety/depressive disorders and 20% reporting behavioural disorders. In contrast, France reported 62% with behavioural disorders as the primary diagnosis and 25% with anxiety and/or depressive disorders. Belgium, Croatia, Italy and The Netherlands were more evenly split between the two largest diagnostic groups. In terms of the other diagnostic groups, Croatia and Italy had more young people with psychotic disorders (23% and 16.4%, respectively) than the other countries combined.

**Fig. 1:**
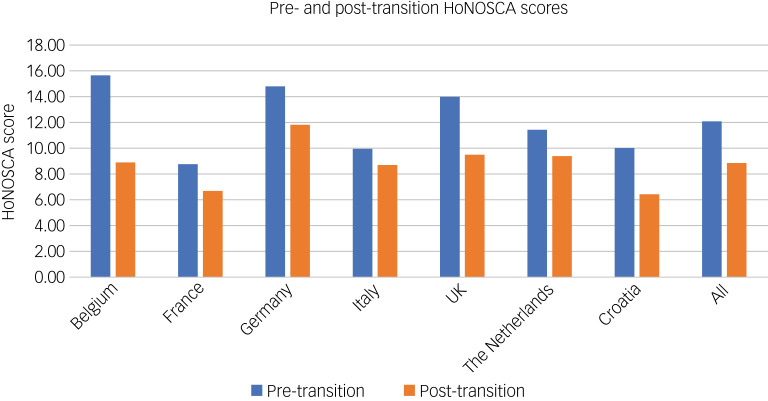
HoNOSCA scores pre- and post-boundary. HoNOSCA, Health of the Nation Outcome Scales for Children and Adolescents.

Figure 1 presents HoNOSCA scores pre- and post-service boundary (the information in this and all subsequent figures is presented in further detail in the Supplementary Material). Pre-boundary, the sample mean HoNOSCA score was 12.09 (s.d. 6.93), with scores ranging from zero (best possible mental health state) to 40 (worst possible mental health state). On average, Belgium (15.64), Germany (14.80) and the UK (13.99) had worse mental health states than France (8.75), Italy (9.95), Croatia (10.02) and The Netherlands (11.42). France, Croatia and Italy had zero young people with HoNOSCA scores <30. After the service boundary, there were improvements in HoNOSCA scores across all countries, particularly in Belgium. The mean reduction (improvement) in HoNOSCA score for all countries post-boundary was 3.25.

The mean severity tertile HoNOSCA scores for each country pre-boundary are shown in Table 1. HoNOSCA is an integer score, with higher levels indicating worse severity. Therefore, the process of constructing tertiles divided participants into three levels of increasingly high HoNOSCA scores that were each roughly, but not exactly, a third of participants. Belgium and Germany reported patients with the worst mental health functioning across tertiles, whereas France and Italy reported patients with the best mental health functioning across tertiles.

**Table 1:** Health of the Nation Outcome Scales for Children and Adolescents tertile mean scores

	Severity tertile (*n*)	Mean pre-boundary HoNOSCA score	95% CI lower	95% CI upper
The Netherlands	Low (*n* = 38)	7.07	6.34	7.81
	Medium (*n* = 39)	13.74	13.15	14.34
	High (*n* = 33)	22.24	20.29	24.20
UK	Low (*n* = 40)	4.60	3.87	5.33
	Medium (*n* = 45)	11.64	11.12	12.17
	High (*n* = 33)	19.39	17.68	21.11
Germany	Low (*n* = 39)	7.95	6.95	8.95
	Medium (*n* = 32)	14.81	14.22	15.40
	High (*n* = 33)	22.88	21.25	24.51
France	Low (*n* = 30)	3.10	2.34	3.86
	Medium (*n* = 23)	9.09	8.48	9.70
	High (*n* = 23)	15.78	14.50	17.07
Italy	Low (*n* = 62)	3.97	3.52	4.42
	Medium (*n* = 64)	9.38	8.95	9.80
	High (*n* = 57)	17.10	16.08	18.13
Belgium	Low (*n* = 38)	9.00	7.86	10.14
	Medium (*n* = 30)	16.13	15.68	16.59
	High (*n* = 27)	24.44	22.64	26.25
Croatia	Low (*n* = 24)	5.17	4.18	6.15
	Medium (*n* = 14)	10.14	9.64	10.64
	High (*n* = 14)	18.21	15.65	20.78
All	Low (*n* = 251)	4.97	4.69	5.26
	Medium (*n* = 273)	11.93	11.70	12.17
	High (*n* = 214)	20.62	19.99	21.25

HoNOSCA, Health of the Nation Outcome Scales for Children and Adolescents.

### Service utilisation

Differences in HoNOSCA are reflected in the number of young people using in-patient services before the service boundary (Table 2). Belgium (35%) and Germany (38%) had the most young people engaged with in-patient services. Conversely, Italy and France had just 13% and 14% in contact with in-patient services, respectively. Across the sample, 19% had contact with in-patient services in the 6 months before the service boundary. At the post-boundary assessment, the numbers in contact with any in-patient care had fallen across the sample, with just 8% still in contact with in-patient services in the 6 months before the assessment time point. Germany and Belgium continued to have the highest proportion of patients using in-patient services, albeit less than half of their pre-boundary level. The numbers of young people contacting in-patient services also decreased from pre- to post-boundary across all other countries.

**Table 2:** Number of young people using services pre- and post-boundary between child/adolescent and adult mental health services

Country	Service use pre-boundary			Service use post-boundary		
	Number receiving in-patient care	Number receiving out-patient care	Number receiving community care	Number receiving in-patient care	Number receiving out-patient care	Number receiving community care
Belgium	33 (35%)	67 (71%)	53 (65%)	8 (11%)	18 (24%)	56 (76%)
France	9 (14%)	44 (68%)	42 (79%)	4 (8%)	22 (46%)	32 (67%)
Germany	34 (38%)	46 (53%)	55 (75%)	10 (18%)	19 (35%)	42 (76%)
Italy	22 (13%)	123 (74%)	131 (84%)	9 (6%)	70 (49%)	88 (62%)
The Netherlands	10 (9%)	37 (32%)	87 (83%)	6 (7%)	23 (26%)	62 (68%)
UK	12 (11%)	74 (68%)	97 (93%)	7 (9%)	27 (34%)	59 (78%)
Croatia	13 (27%)	35 (76%)	27 (75%)	1 (2%)	20 (44%)	20 (44%)
Total	133 (19%)	426 (62%)	492 (81%)	45 (8%)	199 (37%)	359 (67%)

Approximately 62% of young people had contact with any out-patient service in the 6 months before the pre-boundary assessment. This was not consistent across countries. The UK, Italy, Belgium, Croatia and France all had over 66% of patients in contact with out-patient services. For Germany and The Netherlands, the figures were 53% and 32%, respectively. Fifteen months later, the numbers contacting out-patient services had dropped across all countries, to 37%. Belgium, the UK and Croatia saw the largest decreases in the numbers of young people contacting out-patient services (decreases of 47%, 34% and 32%, respectively). France, Germany and Italy also saw considerable decreases in those contacting out-patient services. This change was the least pronounced in The Netherlands, which fell by 6%; however, it was starting from a far lower baseline level than the other countries.

Most patients (81%) were in contact with a community healthcare service in the 6 months before baseline assessment, ranging across countries from 65% (Belgium) to 93% (the UK). Post-service boundary, the proportion of young people who had used any community healthcare service within the previous 6 months decreased to 67%. Croatia, France, Italy, The Netherlands and the UK all experienced a reduction of over 10% for young people in contact with community services. However, Belgium and Germany both saw modest increases in community care contacts from pre- to post-boundary. This may be a consequence of the higher severity of mental disorder and in-patient use pre-boundary, with some of these young people transitioning to community services at the service boundary.

### Costs of care

**Fig. 2:**
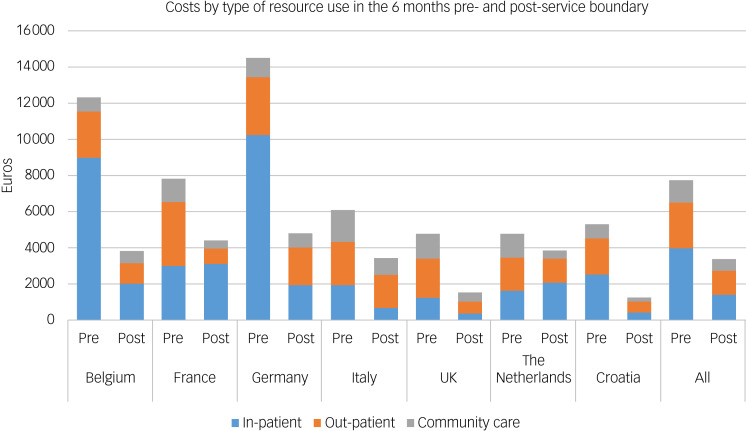
Cost component before and after the service boundary.

Figure 2 shows the healthcare costs before and after the service boundary for each country. All countries saw substantial falls in cost across the service boundary, from € 7761 to € 3376 on average (a reduction of 57%). The largest absolute falls in cost were in Germany and Belgium, the two countries with the most severely ill young people, which saw large falls in in-patient care cost following the service boundary. Large falls in terms of percentage change were seen across all countries (reductions ranging from 43% to 76%), except for The Netherlands (a reduction of 19%). Examining the sample, the main cost driver before the service boundary was in-patient care costs, which accounts for approximately 50% of the total costs. Out-patient costs were the next biggest driver, followed by community care costs. After the service boundary, all cost components fell, with total cost falling by over 50%. This reflects the fall in the number of people engaging with services, and is consistent with the improvement in HONOSCA scores. In-patient care was by far the largest driver of cost in Belgium and Germany, the two countries with the worst mental health functioning scores before the service boundary. However, healthcare costs in Croatia, France, Italy, the UK and The Netherlands were much more evenly spread across the different key cost components.

The costs presented in Fig. 2 are pooled across study arms and include imputed values for missing data. Supplementary Table 6 compares these with costs with missing data excluded, and also presents a breakdown of costs by arm. Pre-boundary, costs were reported by 627 (81%) of participants, and the complete case mean was within 2% of the imputed mean. Post-boundary, costs were reported by 505 (65%) of participants, and the difference between complete case and imputed means was 7%. Supplementary Table 6 also shows that the fall in costs pre- versus post-boundary was comparable between arms. Costs fell by 55% in the intervention arm compared with 59% in the control arm, and there was no statistically significant difference in costs between arms either pre- or post-boundary.

**Fig. 3:**
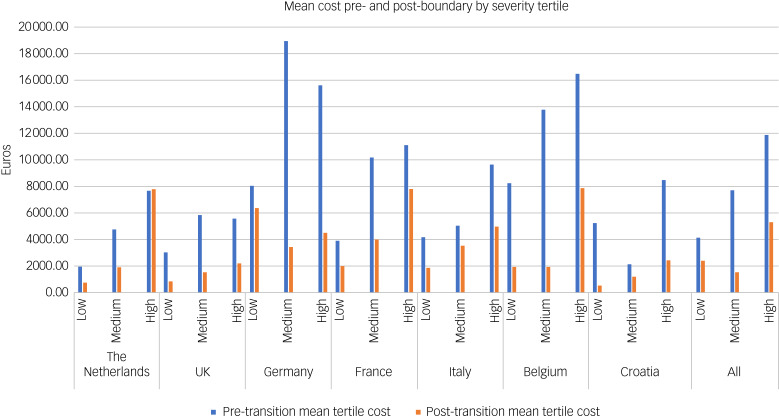
Mean costs by severity tertile.

Figure 3 presents the mean costs for each HoNOSCA tertile for each country before and after the service boundary. Unsurprisingly, when looking at the whole sample, we see that those with the worst mental health (high tertile) have the highest costs, nearly three times the amount (€11 923 *v.* €4199) of those in the lowest severity tertile in the 6 months before the service boundary. At the post-service boundary assessment, those in the high-severity tertile pre-boundary experienced twice the costs (€5311) of those in the low-severity tertile (€2402). Pre-boundary, the gradient of costs across severity was consistent in all countries except France and Croatia. In France, the medium- and high-severity tertiles were equally costly, with a large degree of uncertainty, whereas in Croatia, the low-severity tertile accrued more costs than the medium-severity tertile. Post-service boundary, this pattern was reflected in all countries except for Germany, which was more evenly distributed across tertiles; however, uncertainty was pervasive. For all countries except The Netherlands, costs for all tertiles were lower following the service boundary than they were before the service boundary. In The Netherlands, those in the most severe tertile were roughly equally as costly following the service boundary as before, but there is a high degree of uncertainty in these estimates.

**Fig. 4:**
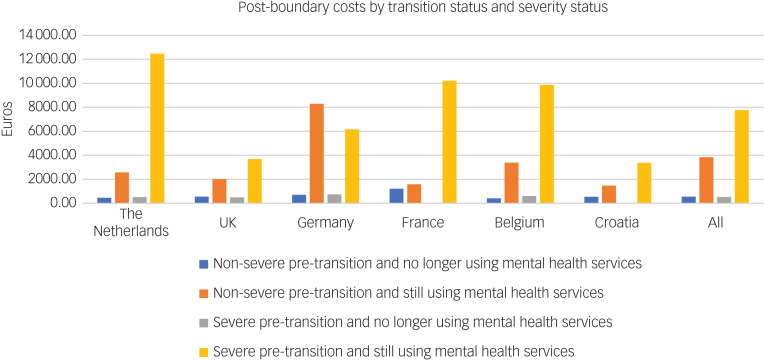
Cost by severity status and mental health engagement post-boundary (engagement data were not collected in Italy).

Figure 4 shows the costs post-boundary according to whether young people were still engaged in mental health services, stratified by the binary ‘severest’ variable. Those who were most severely ill and still engaged with mental health services accrued the highest costs. This was consistent across all countries. Those who were less severely ill but were still engaged with mental health services accrued greater costs than those who were severely ill but had disengaged with mental health services.

## Discussion

We present, to the best of our knowledge, the first multinational analysis of resource use by young people crossing the transition boundary from paediatric to adult mental healthcare. The National Institute for Health and Care Excellence guidelines on Transition from Paediatric to Adult Health Care did not find a single analysis of transitional care from a health economic perspective.^25^ The MILESTONE study was the first ever randomised controlled trial of a transitional care intervention, and the first to explore how transition affects service use. We therefore believe that our work fills an important gap in the evidence base for policy makers and researchers.

### Change in costs from pre- to post-boundary

Costs were substantial pre-boundary. There was a large fall in the proportion of young people engaging with healthcare services post-transition, and in turn, costs reduced (−57%) on average for all countries. Several possible explanations exist for this, including (a) that young people's mental health improved sufficiently enough not to need further care; (b) there was a lack of space or availability of services to transition to, causing undesired discharge from mental health services and (c) young people disengaged with healthcare services, or relocated to an alternative (e.g. the voluntary sector). We cannot prove causality within this study for any of these possibilities, although HoNOSCA scores improved at 15 months, indicating that some young people's mental health may have improved sufficiently for them not to require services post-boundary. For some, this is likely a consequence of increasing independence, ‘growing out’ of certain illnesses – as evidenced elsewhere^26^ – or simply regression to the mean (if they were experiencing acutely severe need at the time of recruitment). However, respondents were recruited irrespective of acute symptom presentation, and qualitative interviews with a subset of participants from the UK demonstrated that lack of space/availability for care post-boundary is still a factor for a significant number of individuals.^2^ This may have implications for long-term health and costs, whereby these young people theoretically could re-engage with health services in the future in a worse state and generate a larger cost to society than had they continued engagement with services.^18^

### Cost components pre- and post-boundary

The main cost driver pre-transition was in-patient care, comprising approximately 50% of costs across the sample. At follow-up, costs were more broadly split across healthcare types, with in-patient (42%) and out-patient (39%) care accounting for a similar proportion of costs post-service boundary. This pattern was not consistent across countries. France, The Netherlands, the UK and Italy all had out-patient costs in excess of in-patient costs pre-service boundary, whereas Belgium and Germany had over four times more in-patient costs than out-patient costs. This could be explained by the different case mixes between countries, where Germany and Belgium had the most severely ill young people pre-boundary. In-patient services are extremely costly and, consequently, these two countries increase the sample's mean in-patient costs. It may also be symptomatic of the differences in how healthcare services are both funded and structured in different countries. For example, Germany and Belgium have far more paediatric beds per head than the UK and Italy.^27^ Likewise, the different structures of healthcare funding may lead to different levels of access for complex care. For example, compared with other European countries, Germany has low levels of unmet need, suggesting that young people have access to care that may not be available in other countries,^28^ and the presence of cost-sharing may reduce the numbers engaged with services.^29^

Post- boundary, most countries experienced reductions in all cost components. The largest decreases were seen in Belgium and France's in-patient care costs. This may be a consequence of resolution of mental health symptoms as evidenced by improved HoNOSCA scores, or disengagement with services. The Netherlands was the outlier, with a small increase in in-patient costs post-boundary compared with pre-boundary despite a slight fall in the numbers engaging with in-patient services. The Netherlands had low rates of in-patient use pre-boundary, and this pattern can be explained by two individuals who required acute psychiatric care for nearly the entire follow-up period and accounted for over 65% of the in-patient healthcare costs for The Netherlands sample, consistent with wide confidence intervals for these estimates.

### Changes in cost according to severity status

There appeared to be a clear pattern in terms of the association with severity status and healthcare costs. Pre- boundary, those in the most severe tertile accrued costs of €11 923 compared with €4199 for those in the low-severity tertile. In all countries except Germany, those with worse mental health states before the service boundary typically accrued substantially greater costs post-transition. It can be expected that those with more severe illnesses require more healthcare in both the short and medium term, as has been found elsewhere in certain condition-specific contexts.^16,30^ Germany saw large decreases in costs in the medium- and high-severity tertiles, whereas the low-severity tertile remained unchanged. An explanation could be that young people engaged with in-patient services pre-boundary were discharged at the service boundary, whereas those in contact with out-patient and community services remained engaged post-transition. This could be seen as either a failure of transition services for in-patients or a success in resolving young people's mental health issues. This is shown in Supplementary Table 8, where young people with non-severe illness in Germany who remained in contact with mental health services post-service boundary accrued high costs. Alternatively, it may be that in-patients were discharged to out-patient care. It should, however, be noted that there was a large amount of uncertainty around these estimates, and so it may simply be a consequence of variability in a small sample.

### Engagement with mental health services and costs

For the subsample of countries where transition destination was known, those who remained in contact with mental health services accrued the most healthcare costs. This was particularly the case for those who were in a severe mental health state at baseline. Even those in the low severity group who remained engaged with services accrued more costs than those in the higher severity group who did not. This suggests that once young people disengage with mental healthcare services, they accrue relatively little costs compared with those that continue engagement with services. Although this may seem beneficial from a short-term cost perspective, there is the issue that some of these individuals may need care but are not able to access it post-transition, as reported by Appleton et al.^2^

### Impact of the MILESTONE intervention

We found no evidence to suggest that the intervention had a substantial impact on costs post-boundary. The MILESTONE intervention was a decision support tool for clinicians aimed at improving decisions around post-boundary care and communication between services. Since this might have affected both decisions to continue care and decisions not to, it was possible *a priori* that the intervention could have either increased or reduced costs. In practice, the trial demonstrated modest improvements in participant mental health and had limited impact on clinical outcomes.^19^ This reflects the challenges to improving complex systems: although our intervention improved communication and participant satisfaction, it could not address challenges such as capacity in adult services. As there is limited evidence that the intervention affected service use, we argue that it was appropriate to pool costs across arms.

### Limitations and future research

Although this paper benefits from a rich data-set across several European countries, several limitations exist. The type of services recruited across countries differed, restricting intercountry comparability of healthcare costs. Thus, we can only talk in general terms relating to possible patterns. For example, those young people recruited in Belgium and Germany had more severe illness than their Italian counterparts, and we would therefore expect their costs to differ. We therefore cannot compare the magnitude of costs between countries in a meaningful way, and instead focus on what happened pre- and post-boundary, and compare within countries and as a pooled sample. We were unable to meaningfully analyse cost data from one country (Ireland), as they recruited just 21 young people. Service destination data was missing for all Italian young people. For other variables, although missing data were relatively limited before the service boundary, it was substantial at follow-up. This was not surprising given the challenges of data collection in this context. Consequently, we relied on multiple imputation, which assumes data is missing at random. This may not have been the case if young people missed appointments with the research team because of an increase in severity of mental illness. Given the sample available, we cannot make definitive statements of statistical significance relating to patterns of healthcare usage, as the trial was not powered for this purpose. This reflects both the sample size and the nature of cost data, which tends to be highly variable and skewed, as was certainly the case in our sample. Likewise, the patterns identified are associations and do not provide evidence of causal pathways.

We used a one-country pooled costing approach because of the quality of unit costs that exist in the UK. If high-quality, up-to-date and accessible unit costs existed for all countries, then a split-country costing approach would be preferable. In certain countries, such as The Netherlands, there is a blurred line between certain out-patient and community care services, and so the relative drivers of cost may be inaccurate when considering the exact splits for out-patient and community care. A further challenge was that, unless services were specifically targeted at young people, separate unit costs were not available for adult versus adolescent patients. Finally, although the second time point is considered ‘post-boundary’, it was possible for young people to not necessarily transfer out of CAMHS. Thus, not all young people necessarily transitioned to AMHS or were discharged.

Costs associated with healthcare in young people with mental health issues are large. We may interpret the fall in costs from pre- to post-boundary as being a success story, especially when considering the overall improvements in HoNOSCA score. However, although some resolution of mental health problems appears to take place,^2^ there may be unmet need following transition. We are unable to say within this study what proportion of reductions in cost are attributable to either unmet need or mental health improvement. Examining whether any unmet need manifests itself in terms of long-term costs and worse health outcomes should be a priority for future research.

## Supporting information

Canaway et al. supplementary material Canaway et al. supplementary material

## Data Availability

The code used to analyse reported data is available from the open-access repository Zenodo (DOI: 10.5281/zenodo.8007712). The data-sets generated and analysed during the current study are not publicly available due to restrictions imposed by participant consent forms, but are available from the corresponding author, J.M., on reasonable request. All research materials used for this study are shared with the MILESTONE study and can be obtained as part of the main study publication (https://doi.org/10.1017/S0033291721003901) or by contacting the corresponding author of that publication.

## References

[1] Singh SP, Tuomainen H. Transition from child to adult mental health services: needs, barriers, experiences and new models of care. World Psychiatry 2015; 14(3): 358–61. 26407794 10.1002/wps.20266 PMC4592661

[2] Appleton R, Elahi F, Tuomainen H, Canaway A, Singh SP. “I'm just a long history of people rejecting referrals” experiences of young people who fell through the gap between child and adult mental health services. Eur Child Adolesc Psychiatry 2021; 30(3): 401–13. 32274589 10.1007/s00787-020-01526-3 PMC8019413

[3] Butterworth S, Singh SP, Birchwood M, Islam Z, Munro ER, Vostanis P, et al. Transitioning care-leavers with mental health needs: ‘they set you up to fail!’. Child Adolesc Ment Health 2017; 22(3): 138–47. 32680381 10.1111/camh.12171

[4] Singh SP, Paul M, Ford T, Kramer T, Weaver T, McLaren S, et al. Process, outcome and experience of transition from child to adult mental healthcare: multiperspective study. Br J Psychiatry 2010; 197(4): 305–12. 20884954 10.1192/bjp.bp.109.075135

[5] Singh SP, Tuomainen H, Bouliotis G, Canaway A, Girolamo GD, Dieleman GC, et al. Effect of managed transition on mental health outcomes for young people at the child–adult mental health service boundary: a randomised clinical trial. Psychol Med 2023; 53(6): 2193–204. 37310306 10.1017/S0033291721003901 PMC10123823

[6] Appleton R, Connell C, Fairclough E, Tuomainen H, Singh SP. Outcomes of young people who reach the transition boundary of child and adolescent mental health services: a systematic review. Eur Child Adolesc Psychiatry 2019; 28(11): 1431–46. 30850925 10.1007/s00787-019-01307-7 PMC6800846

[7] Hovish K, Weaver T, Islam Z, Paul M, Singh SP. Transition experiences of mental health service users, parents, and professionals in the United Kingdom: a qualitative study. Psychiatr Rehabil J 2012; 35(3): 251–7. 22246124 10.2975/35.3.2012.251.257

[8] Singh SP, Paul M, Ford T, Kramer T, Weaver T. Transitions of care from child and adolescent mental health services to adult mental health services (TRACK study): a study of protocols in Greater London. BMC Health Serv Res 2008; 8: 135. 18573214 10.1186/1472-6963-8-135 PMC2442433

[9] Embrett MG, Randall GE, Longo CJ, Nguyen T, Mulvale G. Effectiveness of health system services and programs for youth to adult transitions in mental health care: a systematic review of academic literature. Adm Policy Ment Health 2016; 43(2): 259–69. 25708229 10.1007/s10488-015-0638-9

[10] Santosh P, Adams L, Fiori F, Davidović N, de Girolamo G, Dieleman GC, et al. Protocol for the development and validation procedure of the managing the link and strengthening transition from child to adult mental health care (MILESTONE) suite of measures. BMC Pediatr 2020; 20: 167. 32299401 10.1186/s12887-020-02079-9 PMC7161143

[11] Waldmann T, Stiawa M, Dinc Ü, Saglam G, Busmann M, Daubmann A, et al. Costs of health and social services use in children of parents with mental illness. Child Adolesc Psychiatry Ment Health 2021; 15(1): 10. 33610177 10.1186/s13034-021-00360-y PMC7897390

[12] Beecham JK, Green J, Jacobs B, Dunn G. Cost variation in child and adolescent psychiatric inpatient treatment. Eur Child Adolesc Psychiatry 2009; 18(9): 535–42. 19288167 10.1007/s00787-009-0008-9

[13] Knapp M, Ardino V, Brimblecombe N, Evans-Lacko S, Iemmi V, King D, et al. Youth Mental Health: New Economic Evidence. Personal Social Services Research Unit, London School of Economics and Political Science, 2016 (https://www.pssru.ac.uk/pub/5160.pdf).

[14] Brimblecombe N, Knapp M, Murguia S, Mbeah-Bankas H, Crane S, Harris A, et al. The role of youth mental health services in the treatment of young people with serious mental illness: 2-year outcomes and economic implications. Early Interv Psychiatry 2017; 11(5): 393–400. 26332590 10.1111/eip.12261

[15] Melnyk BM. Reducing healthcare costs for mental health hospitalizations with the evidence-based COPE program for child and adolescent depression and anxiety: a cost analysis. J Pediatr Health Care 2020; 34(2): 117–21. 31615687 10.1016/j.pedhc.2019.08.002

[16] Beecham J, Chadwick O, Fidan D, Bernard S. Children with severe learning disabilities: needs, services and costs. Child Soc 2002; 16(3): 168–81.

[17] Barrett B, Byford S. ‘Costs and outcomes of an intervention programme for offenders with personality disorders’: correction. Br J Psychiatry 2012; 200(6): 514. 10.1192/bjp.bp.109.068643 22361021

[18] Scott S, Knapp M, Henderson J, Maughan B. Financial cost of social exclusion: follow up study of antisocial children into adulthood. BMJ 2001; 323(7306): 191. 11473907 10.1136/bmj.323.7306.191 PMC35269

[19] Singh SP, Tuomainen H, Girolamo GD, Maras A, Santosh P, McNicholas F, et al. Protocol for a cohort study of adolescent mental health service users with a nested cluster randomised controlled trial to assess the clinical and cost-effectiveness of managed transition in improving transitions from child to adult mental health services. BMJ Open 2017; 7(10): e016055. 10.1136/bmjopen-2017-016055 PMC5652531 29042376

[20] Chisholm D, Knapp MRJ, Knudsen HC, Amaddeo F, Gaite L, van Wijngaarden B, et al. Client socio-demographic and service receipt inventory – European version: development of an instrument for international research: ePSILON study 5. Br J Psychiatry 2000; 177(S39): S28–33. 10.1192/bjp.177.39.s28 10945075

[21] Beecham J, Knapp M. Costing psychiatric interventions. In Measuring Mental Health Needs 2nd edn (ed G Thornicroft): 200–24. Gaskell, 2001.

[22] Oppong R, Jowett S, Roberts TE. Economic evaluation alongside multinational studies: a systematic review of empirical studies. PLoS One 2015; 10(6): e0131949. 26121465 10.1371/journal.pone.0131949 PMC4488296

[23] Sterne JAC, White IR, Carlin JB, Spratt M, Royston P, Kenward MG, et al. Multiple imputation for missing data in epidemiological and clinical research: potential and pitfalls. BMJ 2009; 338: b2393. 19564179 10.1136/bmj.b2393 PMC2714692

[24] StataCorp. Stata Statistical Software: Release 16. StataCorp, 2019 (https://www.stata.com/).

[25] Willis ER, McDonagh JE. Transition from children's to adults’ services for young people using health or social care services (NICE guideline NG43). Arch Dis Child Educ Pract 2018; 103(5): 253–6. 10.1136/archdischild-2017-313208 29269436

[26] Patton GC, Coffey C, Romaniuk H, Mackinnon A, Carlin JB, Degenhardt L, et al. The prognosis of common mental disorders in adolescents: a 14-year prospective cohort study. Lancet 2014; 383(9926): 1404–11. 24439298 10.1016/S0140-6736(13)62116-9

[27] Signorini G, Singh SP, Boricevic-Marsanic V, Dieleman G, Dodig-Ćurković K, Franic T, et al. Architecture and functioning of child and adolescent mental health services: a 28-country survey in Europe. Lancet Psychiatry 2017; 4(9): 715–24. 28596067 10.1016/S2215-0366(17)30127-X

[28] The Commonwealth Fund. International Health Care System Profiles: Germany. The Commonwealth Fund, 2020 (https://www.commonwealthfund.org/international-health-policy-center/countries/germany).

[29] Lopes FV, Riumallo Herl CJ, Mackenbach JP, Van Ourti T. Patient cost-sharing, mental health care and inequalities: a population-based natural experiment at the transition to adulthood. Soc Sci Med 2022; 296: 114741. 35144223 10.1016/j.socscimed.2022.114741

[30] Romeo R, Knapp M, Scott S. Economic cost of severe antisocial behaviour in children—and who pays it. Br J Psychiatry 2006; 188(6): 547–53. 16738345 10.1192/bjp.bp.104.007625

