# Supplementary material for: Healthcare costs for young people transitioning the boundary between child/adolescent and adult mental health services in seven European countries: results from the MILESTONE study
Source: BJPsych Open. 2023 Sep 26;9(5):e175. doi: 10.1192/bjo.2023.559 (PMC10617498; doi:10.1192/bjo.2023.559)
Supplement: Canaway et al. supplementary material [file S2056472423005598sup001.docx]

# Appendix

Supplementary materials:

Table S1: Types of care within each care category

| **Inpatient Care categories** | **Unit Cost**  € | **Outpatient Care** | **Unit Cost**  € | **Community Care** | **Unit Cost**  € |
| --- | --- | --- | --- | --- | --- |
| Acute psychiatric ward | 466.56 | Psychiatric outpatient | 327.95 | GP | 42.73 |
| Psychiatric rehabilitation ward | 1094.47 | Other hospital outpatient appointment | 138.40 | Psychiatrist | 124.72 |
| Long-stay psychiatric ward | 466.56 | Day hospital | 411.13 | Paediatrician | 42.73 |
| Emergency crisis center | 1094.47 | Accident and Emergency (without overnight stay) | 150.23 | Psychologist | 115.49 |
| Accident and Emergency (overnight stay) | 178.92 | Other outpatient facilities | 138.40 | District nurse | 45.26 |
| General medical ward | 264.92 |  |  | Community psychiatric nurse / case manager | 61.94 |
| Paediatric ward | 787.61 |  |  | Social worker | 68.14 |
| Residential Rehab Centre | 516.22 |  |  | Occupational therapist | 48.50 |
| Other inpatient Care | 264.42 |  |  | Home help / care worker | 25.41 |
|  |  |  |  | Addition keyworker / community drug and alcohol service worker | 68.14 |
|  |  |  |  | Other community services | 124.72 |

Table S2: Missing Data

| Variable | Missing | Total | % Missing |
| --- | --- | --- | --- |
| Inpatient costs pre-boundary | 85 | 772 | 11 |
| Inpatient costs post-boundary | 235 | 772 | 30 |
| Outpatient costs pre-boundary | 133 | 772 | 17 |
| Outpatient costs post-boundary | 260 | 772 | 34 |
| Community care costs pre-boundary | 94 | 772 | 12 |
| Community care costs post-boundary | 240 | 772 | 31 |
| HoNOSCA pre-boundary | 34 | 772 | 4 |
| HoNOSCA post-boundary | 213 | 772 | 28 |
| Ethnicity | 87 | 772 | 11 |
| Transition destination | 277 | 772 | 36 |
| Primary diagnosis | 25 | 772 | 3 |

Table S3: Primary diagnoses by country

|  | Belgium  (n = 94) | Croatia  (n = 52) | France  (n = 77) | Germany  (n=77) | Italy  (n= 189) | Netherlands  (n = 109) | UK  (n = 119) | Total (n = 747*) |
| --- | --- | --- | --- | --- | --- | --- | --- | --- |
| Anxiety and/or Depressive disorders | 34 | 18 | 19 | 47 | 59 | 37 | 64 | 278 |
|  | 36% | 35% | 25% | 44% | 31% | 34% | 54% | 37% |
| Behavioural disorders | 38 | 16 | 48 | 21 | 71 | 49 | 21 | 264 |
|  | 40% | 31% | 62% | 20% | 38% | 45% | 18% | 35% |
| Feeding and eating disorders | 3 | 0 | 8 | 9 | 18 | 5 | 11 | 54 |
|  | 3% | 0% | 10% | 8% | 10% | 5% | 9% | 7% |
| Psychotic disorders | 4 | 12 | 0 | 9 | 31 | 2 | 6 | 64 |
|  | 4% | 23% | 0% | 8% | 16% | 2% | 5% | 9% |
| Trauma and stressor-related disorders | 11 | 4 | 1 | 9 | 5 | 9 | 11 | 50 |
|  | 12% | 8% | 1% | 8% | 3% | 8% | 9% | 7% |
| Other | 4 | 2 | 1 | 12 | 5 | 7 | 6 | 37 |
|  | 4% | 4% | 1% | 11% | 3% | 6% | 5% | 5% |

NB. Individuals can have more than one primary diagnosis. *25 missing

Table S4: HoNOSCA pre and post service boundary (SB) by country (cf fig 1 in main paper)

| Pre-SB | | | | | |
| --- | --- | --- | --- | --- | --- |
|  | Obs | Mean | Std. dev. | Min | Max |
| Belgium | 95 | 15.64 | 7.15 | 1 | 34 |
| Croatia | 52 | 10.02 | 6.10 | 0 | 26 |
| France | 76 | 8.75 | 5.72 | 0 | 23 |
| Germany | 104 | 14.80 | 7.04 | 0 | 35 |
| Italy | 183 | 9.95 | 5.92 | 0 | 28 |
| UK | 110 | 13.99 | 7.01 | 2 | 40 |
| Netherlands | 118 | 11.42 | 6.57 | 0 | 38 |
| Total | 738 | 12.09 | 6.93 | 0 | 40 |
| Post-SB | | | | | |
|  | Obs | Mean | Std. dev. | Min | Max |
| Belgium | 65 | 8.89 | 5.48 | 0 | 23 |
| Croatia | 47 | 6.43 | 5.64 | 0 | 22 |
| France | 69 | 6.68 | 4.95 | 0 | 22 |
| Germany | 59 | 11.81 | 7.85 | 0 | 30 |
| Italy | 141 | 8.70 | 5.40 | 0 | 25 |
| Netherlands | 90 | 9.49 | 6.50 | 0 | 31 |
| UK | 88 | 9.39 | 6.58 | 0 | 26 |
| All | 559 | 8.84 | 6.20 | 0 | 31 |

Table S5: Healthcare costs pre and post service boundary (cf fig 2 in main paper)

|  | Time point | Inpatient care costs € | Outpatient care costs € | Community Care costs  € | Total  Costs (95% CI)  € | % Change |
| --- | --- | --- | --- | --- | --- | --- |
| Belgium  (n = 97) | Pre | 8976.96 | 2525.35 | 819.24 | 12321.55  (8754-15889) | -69% |
|  | Post | 1998.31 | 1149.60 | 648.94 | 3796.85  (277-7316) |  |
| France  (n = 79) | Pre | 3002.47 | 3528.03 | 1287.67 | 7818.17  (3373-12264) | -44% |
|  | Post | 3087.60 | 873.06 | 425.44 | 4386.11  (772-8000) |  |
| Germany  (n = 109) | Pre | 10244.70 | 3192.04 | 1023.04 | 14459.77  (9846-19073) | -67% |
|  | Post | 1899.61 | 2109.35 | 783.76 | 4792.71  (2085-7500) |  |
| Italy  (n = 190) | Pre | 1965.42 | 2407.20 | 1738.09 | 6110.71  (4013-8208) | -43% |
|  | Post | 704.96 | 1765.64 | 988.58 | 3459.18  (2231-4687) |  |
| UK  (n = 127) | Pre | 1246.28 | 2156.41 | 1370.60 | 4773.29  (2962-6584) | -68% |
|  | Post | 361.72 | 660.82 | 511.23 | 1533.77  (389-2678) |  |
| Netherlands  (n = 118) | Pre | 1629.02 | 1821.95 | 1291.38 | 4742.35  (2691-6793) | -19% |
|  | Post | 2061.65 | 1303.56 | 463.89 | 3829.10  (1092-6566) |  |
| Croatia  (n = 52) | Pre | 2522.61 | 1998.49 | 779.26 | 5300.36  (3120-7481) | -76% |
|  | Post | 439.43 | 599.56 | 208.82 | 1247.80  (0-3319) |  |
| All (n = 772) | Pre | 3989.30 | 2489.31 | 1282.27 | 7760.88  (6625-8897) | -57% |
|  | Post | 1412.98 | 1314.50 | 648.11 | 3375.59  (2517-4234) |  |

|  | **Time Point** | **Imputed pooled costs** | | **Imputed costs – intervention arm** | | **Imputed costs – control arm** | | **Complete case analysis pooled costs** | |
| --- | --- | --- | --- | --- | --- | --- | --- | --- | --- |
|  |  | **N** | **Total Cost €**  **Mean (SE)** | **N** | **Total Cost €**  **Mean (SE)** | **N** | **Total Cost €**  **Mean (SE)** | **N** | **Total Cost €**  **Mean (SE)** |
| **Belgium** | Pre | 97 | 12321.55 (1820.24) | 33 | 11088.1 (2201.1) | 64 | 12799.01 (2379.89) | 84 | 12702.09 (1920.98) |
|  | Post | 97 | 3796.85 (1795.74) | 33 | 7309.7 (4748) | 64 | 2039.1 (467.62) | 72 | 4332.56 (2218.49) |
| **France** | Pre | 79 | 7818.17 (2268.1) | 13 | 2338.72 (901.72) | 66 | 9055.49 (2439.78) | 57 | 7078.61 (2012.2) |
|  | Post | 79 | 4386.11 (1843.8) | 13 | 838.66 (392.32) | 66 | 4843.8 (1719.74) | 48 | 5140.73 (2362.04) |
| **Germany** | Pre | 109 | 14459.77 (2353.83) | 45 | 11116.8 (2047.81) | 64 | 16621.51 (3134.19) | 80 | 14653.64 (2715.16) |
|  | Post | 109 | 4792.71 (1381.28) | 45 | 3560.3 (920.65) | 64 | 6265.03 (1854.7) | 52 | 5697.36 (2354.68) |
| **Italy** | Pre | 190 | 6110.71 (1070.13) | 63 | 12039.24 (2589.05) | 127 | 3138.1 (548.56) | 151 | 6620.69 (1218.02) |
|  | Post | 190 | 3459.18 (626.55) | 63 | 6641.85 (1208.54) | 127 | 1874.08 (229.25) | 136 | 3682.9 (621.45) |
| **UK** | Pre | 127 | 4773.29 (924.03) | 43 | 4690.58 (953.14) | 84 | 5060.98 (1025.19) | 103 | 4888.4 (961.94) |
|  | Post | 127 | 1533.77 (584.04) | 43 | 1872.78 (462.69) | 84 | 1641.73 (245.25) | 71 | 1310.08 (299.42) |
| **Netherlands** | Pre | 118 | 4742.35 (1046.37) | 35 | 1571.9 (241.56) | 83 | 6731.94 (1539.65) | 108 | 3896.68 (775.32) |
|  | Post | 118 | 3829.1 (1396.37) | 35 | 1032.96 (227.18) | 83 | 5704.35 (1943.47) | 84 | 4003.3 (1674.53) |
| **Croatia*** | Pre | 52 | 5300.36 (1112.36) | 0 | NA | 52 | 1247.8 (636.64) | 44 | 5232.23 (1039.27) |
|  | Post | 52 | 1247.8 (636.64) | 0 | NA | 52 | 7760.88 (579.6) | 42 | 982.02 (317.42) |
| **Total** | Pre | 772 | 7760.88 (579.6) | 232 | 7888.78 (891.49) | 540 | 7651.91 (1020.14) | 627 | 7650.77 (350.21) |
|  | Post | 772 | 3375.59 (438.02) | 232 | 3549.76 (716.58) | 540 | 3119.6 (424.88) | 505 | 3616.58 (175.98) |

*Table S6: Comparison of reference costs with costs broken down by study arm and costs calculated without imputation.*

*No participants in Croatia received the intervention.

Table S7: Cost by severity tertile (cf fig 3 in main paper)

|  | Severity | Pre-boundary mean tertile cost (95% CI) € | Post-boundary mean tertile cost (95% CI) € |
| --- | --- | --- | --- |
| Netherlands | Low (n = 38) | 1952.50 (1121-2784) | 766.77 (0-1772) |
|  | Medium (n = 39) | 4776.64 (1221-8333) | 1929.10 (106-3752) |
|  | High (n = 33) | 7708.98 (2082-13336) | 7786.21 (0-15710) |
| UK | Low (n = 40) | 3061.87 (1934-4190) | 850.91 (0-2189) |
|  | Medium (n = 45) | 5909.28 (1713-10106) | 1560.57 (73-3048) |
|  | High (n = 33) | 5563.38 (2173-8954) | 2218.73 (0-5293) |
| Germany | Low (n = 39) | 8082.76 (2473-13692) | 6391.59 (148-12635) |
|  | Medium (n = 32) | 18983.47 (8133-29834) | 3457.50 (0-7512) |
|  | High (n = 33) | 15647.42 (7917-23378) | 4538.48 (786-8291) |
| France | Low (n = 30) | 3926.41 (135-7718) | 1990.35 (0-5354) |
|  | Medium (n = 23) | 10214.16 (0-21554) | 4053.62 (0-10486) |
|  | High (n = 23) | 11159.84 (2155-20165) | 7839.70 (0-17426) |
| Italy | Low (n = 62) | 4173.49 (767-7580) | 1906.50 (343-3470) |
|  | Medium (n = 64) | 5024.85 (2002-8048) | 3542.27 (1469-5615) |
|  | High (n = 57) | 9636.68 (5226-14047) | 5001.10 (2767-7235) |
| Belgium | Low (n = 38) | 8265.50 (2414-14117) | 1940.00 (0-4850) |
|  | Medium (n = 30) | 13824.20 (8297-19352) | 1914.46 (0-4068) |
|  | High (n = 27) | 16508.72 (9164-23853) | 7899.56 (0-19571) |
| Croatia | Low (n = 24) | 5258.32 (2381-8135) | 587.67 (0-1520) |
|  | Medium (n = 14) | 2166.19 (0-5729) | 1193.65 (0-5927) |
|  | High (n = 14) | 8506.59 (3441-13572) | 2433.62 (0-7024) |
| All | Low (n = 263) | 4198.55 (2954-5443) | 2402.12 (1171-3633) |
|  | Medium (n = 218) | 7727.89 (5521-9934) | 2496.46 (1554-3439) |
|  | High (n = 205) | 11923.26 (9514-14332) | 5311.06 (2980-7642) |

Table S8: Cost by severity status and MH engagement post-boundary boundary (cf fig 4 in main paper)

|  | **Non-severe prior and no longer using MH services post boundary (95% CI)** | **Non-severe prior but still using MH services post boundary (95% CI)** | **Severe prior but no longer using MH services post boundary (95% CI)** | **Severe prior but still using MH services post boundary (95% CI)** |
| --- | --- | --- | --- | --- |
| **Netherlands** | 451.09 (0-956) | 2568.52 (166-4971) | 513.97 (0-1212) | 12465.23 (0-26113) |
| **UK** | 546.95 (0-1348) | 2008.46 (671-3346) | 490.02 (0-2027) | 3679.43 (41-7318) |
| **Germany** | 709.12 (0-3217) | 8283.55 (971-15596) | 740.84 (0-5867) | 6165.68 (188-12143) |
| **France** | 1206.09 (0-8956) | 1578.76 (0-6238) | N/A** | 10211.88 (0-23877) |
| **Belgium** | 408.37 (0-2112) | 3389.81 (0-6858) | 599.97 (0-6918) | 9864.06 (25665) |
| **Italy*** | N/A* | N/A* | N/A* | N/A* |
| **Croatia** | 540.21 (0-1988) | 1475.31 (0-8601) | 90.57 (0-480) | 3370.83 (0-11743) |
| **All** | 549.98 (0-1222) | 3835.27 (1861-5810) | 520.47 (0-1192) | 7753.67 (3406-12101) |

*Engagement data was not collected in Italy. **zero observations
